# Residue Elimination Patterns and Determination of the Withdrawal Times of Seven Antibiotics in Eggs of Taihang Chickens

**DOI:** 10.3390/ani14243701

**Published:** 2024-12-22

**Authors:** Huan Chen, Xiajun Wu, Shasha Cui, Yandong Li, Yingli Mu, Jinduo Gao, Huage Liu, Juxiang Liu

**Affiliations:** 1 College of Veterinary Medicine, Hebei Agricultural University, Baoding 071000, China; chenhuantx@163.com (H.C.); muyingli07@163.com (Y.M.); 18333304198@163.com (J.G.); 2 Hebei Provincial Station of Veterinary Drug and Feed, Shijiazhuang 050035, China; hbsswxj@163.com (X.W.); serafina_sha@126.com (S.C.); 15613108352@163.com (Y.L.); 3 Institute of Animal Husbandry and Veterinary Medicine of Hebei Province, Baoding 070066, China

**Keywords:** Taihang chicken, free-range conditions, eggs, residue elimination, withdrawal time

## Abstract

The objective of this study was to examine the residue elimination patterns of seven antibiotics in the eggs of Taihang chickens under free-range conditions and develop suitable withdrawal times (WDTs). A total of 240 healthy Taihang chickens, aged 180 days, were randomly divided into eight groups of 30 birds each. The first seven groups were administered oxytetracycline, chlortetracycline, erythromycin, tylosin, tylvalosin, lincomycin, and tiamulin, respectively, in accordance with the maximum dosages and longest durations of treatment recommended by the Veterinary Pharmacopoeia of the People’s Republic of China. Group 8 served as the control group, and the test period continued until no drug residue could be detected. The results demonstrate that the residues of oxytetracycline, chlortetracycline, tylosin, tylvalosin, and tiamulin throughout the test period were below the maximum residue limits (MRLs) outlined in GB 31650-2019, “National food safety standard-Maximum residue limits for veterinary drugs in foods”. The egg yolk was identified as the target tissue for estimating the withdrawal time periods (WDTs) of these seven drugs. It was thus concluded that the recommended withdrawal time for tiamulin should be 0 days. However, the WDTs of oxytetracycline, chlortetracycline, tylosin, tylvalosin, were 2.8, 0.3, 2.4, and 7.4, respectively, when the upper limit of the 95% confidence interval was found to be lower than the MRLs. It was thus determined that the recommended WDTs for oxytetracycline, chlortetracycline, tylosin, and tylvalosin should be 3, 1, 3, and 8 days, respectively. In contrast, erythromycin and lincomycin exhibited concentrations below the permitted MRLs on days 7 and 8, respectively. However, the upper limits of the 95% confidence intervals for erythromycin and lincomycin were found to be below the MRLs when the WDTs were 11 and 8.9, respectively. This suggests that the recommended WDTs for these two antibiotics should be 11 days and 9 days.

## Simple Summary

Antibiotic residues in eggs can not only threaten human health, but also lead to the development and spread of antibiotic resistance. Therefore, it is important to determine appropriate withdrawal times for antibiotics in laying hens; these have not been reported for Taihang chickens. The objective of this research was to study the residue elimination patterns of seven antibiotics in Taihang chickens eggs under free-range conditions and develop appropriate withdrawal times. According to the results of this study, the recommended withdrawal time of tiamulin in the eggs of Taihang chickens was 0 days. The recommended withdrawal times of oxytetracycline, chlortetracycline, erythromycin, tylosin, tylvalosin, and lincomycin were 3, 1, 11, 3, 8, and 9 days, respectively. The results of this study provide data support for the development of withdrawal times for antimicrobials in Taihang chickens to ensure appropriate medication and food safety. They provide a technical reference for guaranteeing the development of the Taihang chicken industry.

## 1. Introduction

Eggs are the most common and most consumed high-protein product that have high quality and low price in the diets of Chinese people, with an annual per capita consumption of more than 18 kg. The quality and safety of eggs are therefore of great importance to public health. The presence of antibiotic residues in eggs not only poses a threat to human health, but also can facilitate the development and dissemination of antibiotic resistance [1,2]. In light of these considerations, the state has issued a national food safety standard, GB 31650-2019, entitled “National food safety standard: Maximum residue limits for veterinary drugs in foods” [3]. According to this standard, 11 antibiotics are permitted for use during the egg-laying period. These are oxytetracycline, chlortetracycline, tetracycline, erythromycin, tylosin, tylvalosin, lincomycin, tiamulin, neomycin, spectinomycin, and colistin. Oxytetracycline and chlortetracycline are tetracyclines, a group of alkaline broad-spectrum antibiotics produced by *Streptomyces* or semi-synthesised. They are effective against a wide range of Gram-positive and -negative bacteria, *mycoplasmas*, *chlamydia*, and *rickettsiae* [4,5]. The macrolides, which include erythromycin, tylosin, and tylvalosin, are a group of weakly alkaline antibiotics produced or semi-synthesised by *Streptomyces*. They each possess a 14-membered ring lactone structure. Macrolides have been demonstrated to be effective against mycoplasmas and Gram-positive bacteria (such as *Streptococcus* and *Staphylococcus*), but their efficacy against Gram-negative bacteria is limited [6,7,8]. Lincomycin is a member of the lincosamide class, a category of antibiotics derived from *Streptomyces* fermentation broth. Its mechanism of action entails binding to the 50S subunit of the bacterial ribosome, thereby inhibiting protein synthesis. This class of antibiotics has strong antibacterial activity against Gram-positive bacteria and *mycoplasma*, and also has a certain effect on anaerobes, but is not sensitive to most aerobic Gram-negative bacteria [9]. Tiamulin is an antibacterial antibiotic belonging to the truncated Pleurotus class; it is primarily utilised to prevent and treat chronic respiratory disease in chickens. Tiamulin has good antibacterial activity against many Gram-positive bacteria, including most staphylococci and streptococci (except group D streptococci), and also has good antibacterial activity against mycoplasma and *Treponema hyodysenteriae* [10,11]. Residues of oxytetracycline, chlortetracycline, tylvalosin, lincomycin, and tiamulin in eggs are detected directly, while those of erythromycin and tylosin are detected via erythromycin A and tylosin A, respectively.

The growth in the social economy and the enhancement of people’s quality of life have led to an increase in demand for environmentally friendly, natural, and distinctive foods. The Taihang chicken represents a distinctive local breed within the Chinese poultry population. The species is primarily endemic to the province of Hebei, representing a high-quality local resource there. The bird is characterised by an attractive appearance, a compact physique, agility, a thin skin, and well-defined bones. It displays notable characteristics of strong adaptability and disease resistance, tolerance to roughage, and excellent egg and meat quality. It is reared under free-range conditions and constitutes a local breed for both eggs and meat [12,13]. In comparison to contemporary caged chickens, Taihang chickens exhibit distinctive traits, including a high slaughter rate, low abdominal fat rate, and well-developed immune organs [14]. Additionally, their intestines are 0.2–0.3 cm longer than those of ordinary laying hens, and their digestive capacity is more robust. The current number of Taihang chickens raised in the country has reached 20 million, which can provide 192,000 tons of high-quality eggs annually.

The current standards for withdrawal times were formulated for caged commercial laying hens; none have been specified for Taihang chickens. Given the disparate breeding techniques and physiological attributes of various chicken breeds and production methods, the elimination of drug residues naturally differs. This variability impacts the ideal WDTs for different antibiotics. The Taihang chicken represents a distinctive breed in Hebei Province. It is imperative to investigate the elimination patterns of pertinent antibiotics in the Taihang chicken’s body and establish the optimal WDTs. For this reason, we conducted this study with the aim of recommending appropriate WDTs, thereby providing a scientific basis for guiding the safe clinical use of drugs and ensuring the safety of Taihang chicken products.

## 2. Materials and Methods

### 2.1. Animals

Taihang chickens, purchased from Huafeng Leyuan Agricultural and Animal Husbandry Science and Technology Co., Ltd. (Shijiazhuang, China) started laying eggs at 120 days of age and were selected to have a relatively stable egg production at the peak of egg laying (180 days of age; initial body weight: 1530 ± 88 g) [15]. One week prior to the start of the experiment, all the laying hens were uniformly managed, with similar management conditions and controlled microclimate in all the groups of laying hens at the time of the experiment (temperature: 21–22 °C, humidity 65–68%), normal water and feed intake, and a light period of 16 h (from 06:00 to 22:00). The basal diet was a corn–soybean meal-based diet and was a commercial feed from Tianniu Company (Shijiazhuang, China).

### 2.2. Drugs and Reagents

Experimental drugs: oxytetracycline hydrochloride soluble powder (50%), batch number 20220702, Hebei Xiangda Hezhong Biotechnology Co., Ltd. (Shijiazhuang, China); chlortetracycline hydrochloride soluble powder (20%), batch number 20220703; erythromycin thiocyanate soluble powder (5%), batch number 20220906; tylosin tartrate soluble powder (50%), batch number 202202002, Baoding Ji Zhong Pharmaceutical Co., Ltd. (Baoding, China); tylvalosion tartrate soluble powder (20%), batch number 22081401, Zhangjiakou Wanquan District Ketai Biotechnology Co., Ltd. (Zhangjiakou, China); lincomycin hydrochloride soluble powder (10%), batch number 20230043, Hebei Zhenghe Biopharmaceutical Co., Ltd. (Xingtai, China); and tiamulin fumarate soluble powder (45%), batch number 20220538, produced by Hebei Weili Biotechnology Co., Ltd (Shijiazhuang, China).

Antibiotic standards: oxytetracycline, chlortetracycline, erythromycin, tylosin, tylvalosin, lincomycin, and tiamulin (Bepure, Philadelphia, PA, USA).

Reagents: methanol and acetonitrile (chromatographic grade, Merck, Darmstadt, German), distilled water (Watsons, Beijing, China), formic acid (Thermo Fisher Scientific, Waltham, MA, USA), and an Oasis PRIME HLB (500 mg, 6 mL) solid-phase extraction column (Waters, Milford, MA, USA).

Standard stock solution: 10.0 mg of each standard was accurately weighed and prepared as 1 mg/mL of standard stock solution, stored at −18 °C, and protected from light.

### 2.3. Experimental Design

All the animal experiments were conducted at the Animal Experiment Centre of the College of Veterinary Medicine of Hebei Agricultural University and approved by the Animal Ethics Committee of Hebei Agricultural University (approval document number: 20231187; approval date: 20 February 2023). All the experiments were conducted by duly qualified personnel in accordance with the “Standards for the Management of Experimental Animals” of the Ministry of Science and Technology of China. The 240 chickens were randomly divided into 8 groups, with 30 chickens in each group (6 per cage, with 5 replicates). Groups 1–7 were the experimental groups, and the chickens were administered seven different antibiotics: oxytetracycline, chlortetracycline, erythromycin, tylosin, tylvalosin, lincomycin and tiamulin. Group 8 served as the control group. Prior to the commencement of the experiment, the six animals in each cage were numbered. In accordance with the “Guidelines for Veterinary Drug Residue Elimination Test” No. 326 of the Ministry of Agriculture and Rural Affairs (2020), the experimental group was administered the maximum dose and longest course of treatment recommended by the veterinary pharmacopoeia, while the control group was not treated with any antimicrobial drugs. The breeding environments of the eight groups were identical. During the course of the experiment, the chickens were provided with feed that had been produced without the use of antibiotics and had free access to water. The health of the chickens was monitored daily (feed intake, water intake, respiration, mental status, egg production, defecation, etc.), and egg samples were collected for analysis. The experimental group was administered the specified pharmaceutical agents in accordance with the methodology delineated in Table 1 at 8:00 a.m. each day. The tiamulin group was administered for 3 consecutive days, the lincomycin group was administered for 10 consecutive days, and the rest of the test groups were administered for 5 consecutive days; the control group, in contrast, was not administered any antimicrobial. From the initial administrations in the experimental groups, egg samples were collected at 12:00 each day until no drug residues could be detected. Samples of albumen, yolk, and whole egg were taken separately, mixed evenly, and then rapidly cooled and stored at −20 °C. Blank samples were also taken as controls.

### 2.4. Sample Preparation

Drug extraction from eggs was performed as previously described [16], with some modifications. The albumen and yolk were separated and homogenised using a GM200 high-speed dispersing homogeniser (Retsch GmbH, Haan, Germany). After thorough stirring, 2.0 g of each was weighed for subsequent drug extraction. The sample was added to 8 mL of acidified acetonitrile (0.2% formic acid), vortexed for 10 min, sonicated for 10 min, and centrifuged at 12,000 rpm for 10 min. The supernatant was purified on an Oasis PRIME HLB solid-phase extraction column without activation and equilibration; the flow rate was maintained at one drop per second, and the volume was adjusted to 3 mL. Then, 3 mL of the supernatant was evaporated to dryness under a nitrogen stream at 40 °C. The residue was dissolved in 0.6 mL of mobile phase (0.1% formic acid–water–acetonitrile (96:4)) and analysed using LC-MS/MS through a 0.22 μm GHP filter (Pall Corporation, Port Washington, NY, USA).

### 2.5. Liquid Chromatography–Mass Spectrometry Conditions

A Waters Acquity UPLC Xevo TQ-S (Waters, USA) ultra-high-performance chromatograph–tandem mass spectrometer was used.

Chromatographic conditions: Chromatographic column: C18 (HSS T3 column, 50 mm × 2.1 mm (inner diameter), particle size: 1.8 μm). Mobile phase: 0.1% formic acid–water (A) and acetonitrile (B). Gradient elution conditions: 0~2.5min, 98%A, and 2%B; 2.5~4min, 80%A, and 20%B; 4~4.5min, 10%A, and 90%B; 4.5~5min, 98%A, and 2%B. Flow rate: 0.4 mL/min. Column temperature: 30 °C. Injection volume: 2 μL.

Mass spectrometry conditions: Electrospray ion source (ESI+/−); scanning mode: positive ion scanning; mass spectrometry scanning mode: dynamic multiple reaction ion monitoring; tandem mass spectrometer electrospray ion source: ESI+; capillary voltage: 3.00 kV; desolvation temperature: 400 °C; cone voltage: 65 V; desolvation gas flow: 850 L/hr; collision gas flow: 0.15 mL/min. The characteristic conditions for ion reference mass spectrometry are provided in Table 2.

The albumen, yolk, and whole egg from the control group were used as blank samples; after operating according to the steps in Section 2.4, the matrix solutions of the blank samples were obtained. Then, the working standard solution was diluted to make matrix-matched standard solutions with mass concentrations of 0.2, 0.5, 1.0, 5.0, 20, 50, 100, and 200 ng/mL, respectively; and ultra-high-pressure liquid chromatography–tandem triple quadrupole mass spectrometry (UPLC-MS/MS) was performed.

Seven analytes were spiked into three blank samples at concentrations of 5, 10, and 50 μg/kg, and five replicates were prepared for each concentration. Three batches were prepared in duplicate, and the recoveries, intra-day recoveries, inter-day recoveries, and coefficients of variation were calculated.

### 2.6. Method Validation

The analytical method was fully validated in accordance with European Commission Directive 2002/657/EC. A series of concentrations of the seven analytes spiked into the matrix were used to construct calibration curves. The results showed good linearity for the seven analytes in the range from 0.2 to 200 ng/mL. Appropriate amounts of the seven antibiotic standard working solutions were spiked into the blank sample matrix, and all the samples were quantified using the calibration curves. The limits of detection (LODs) and limits of quantification (LOQs) were calculated from the analysis of the blank egg samples spiked with the seven antibiotic standard working solutions, which were the lowest analyte concentrations that produced a chromatographic peak with a signal-to-noise ratio of 3 (LOD) and 10 (LOQ), respectively.

The determination limit (CCα) and detection capacity (CCβ) were calculated as described by Verdon et al. [17]. The accuracy and precision were tested by spiking six blank samples at three concentration levels on three separate days.

## 3. Results

### 3.1. Method Validation 

**Figure 1:**
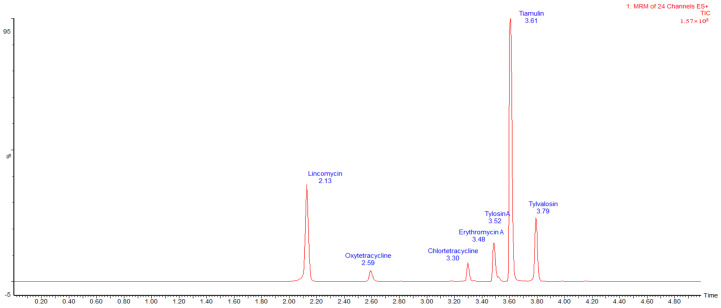
Total ion current chromatogram of seven kinds of antibiotics in egg samples spiked at 50 μg/kg. Note: The erythromycin and tylosin retention times are similar and overlap in the total ion current chromatogram. The quantitative ionogram is shown in Figure S1 of the Supplementary Material.

Under established mass spectrometry conditions, the concentration of the matrix-matched standard solution was determined using UPLC-MS/MS. The regression equation of the matrix standard curve was obtained. The results show that the UPLC-MS/MS method used in this experiment could meet the requirements for detecting seven antibiotics in Taihang chicken eggs. A good linear relationship was shown in the range of 0.2~200 μg/mL, and the correlation coefficient was more than 0.995. The limit of quantification (LOQ) was determined based on where the signal-to-noise ratio of the characteristic ion chromatographic peak was S/N ≥ 10, and the limit of detection (LOD) was determined based on where S/N ≥ 3. The obtained regression equation and correlation coefficient, limit of detection, and limit of quantification are provided in Table 3. Mixed standard working solutions of one, two, and five times the limit of quantification were added to three batches of egg samples, and six parallels were made for each concentration. The recovery of the three groups of added concentrations ranged from 71% to 99%, and the intra-batch and inter-batch precisions were ≤4.3%. The recovery, precision, CCα, and CCβ are shown in Table 4. The total ion current chromatogram for the seven drugs spiked in eggs is shown in Figure 1.

### 3.2. Residue Elimination Rules

#### 3.2.1. Elimination Rules of Oxytetracycline, Chlortetracycline, and Their Main Metabolites

As shown in Figure S2, no oxytetracycline was detected in eggs on the first day of oxytetracycline administration, whereas chlortetracycline residues were detected on the day of administration with a mean concentration of 3.6 μg/kg. During the second to fifth days of continuous administration, the oxytetracycline residue in eggs increased rapidly, peaking on the seventh day in whole eggs and egg yolks, and the concentration of oxytetracycline residue in the albumen peaked on the fifth day. The concentration of oxytetracycline residues in egg yolks was the highest, 65% and 52% higher than in whole eggs and albumens, respectively. The highest chlortetracycline residues in whole eggs, egg yolks, and albumens were all found on day 5. During the medication period, the overall oxytetracycline and chlortetracycline levels in whole eggs, egg yolks, and albumens increased with the number of days of medication.

After drug withdrawal, the oxytetracycline residues in eggs decreased rapidly. The oxytetracycline content in whole eggs, egg yolks, and albumens decreased gradually as the days of drug withdrawal increased, and the elimination rate was positively correlated with the residue. The rate of elimination was faster in the early phase of withdrawal than in the later phase. Within the first 1 to 7 days of drug withdrawal, the chlortetracycline residues decreased rapidly, at a rate of about 5 μg/kg per day. The average residue was 10 μg/kg on day 8. The chlortetracycline elimination rate was rapid for albumen and slow for egg yolk. The pharmacokinetics of veterinary drug residues in egg yolk and albumen showed the same characteristics. The drug residues in albumen reflect the plasma levels and take less time to reach a constant level than the drug residues in egg yolk [18,19]. This difference may be due to multiple factors, including the nature of the drug (lipophilicity and the better permeability of lipophilic egg yolk) and the different physicochemical processes involved in the formation of albumen and egg yolk [20].

Throughout this study, the residues of oxytetracycline and chlortetracycline in eggs were below the maximum residue limit (400 μg/kg) allowed for by GB 31650-2019 “National food safety standard-Maximum residue limits for veterinary drugs in Food” [2]. The residues of oxytetracycline and chlortetracycline in all the eggs in the control group were below the respective detection limits for their methods.

#### 3.2.2. Elimination of Erythromycin, Tylosin, Tylvalosin, and Their Main Metabolites

As shown in Figure S3, erythromycin A was detected in eggs on the first day of administration, whereas tylosin A and tylvalosin were not detected. During the second to fifth day of continuous administration, the residual amounts of erythromycin A, tylosin A and tylvalosin in eggs increased rapidly, with erythromycin A and tylosin A both peaking on the seventh day and tylvalosin on the ninth day.

After drug withdrawal, the erythromycin A residues in eggs decreased rapidly. The residue in whole eggs had decreased to less than 50 µg/kg (43.2 µg/kg) on the fourth day after drug withdrawal, but the residue in yolk did not decrease to less than 50 µg/kg (24 µg/kg) until the seventh day. The residue of tylosin A in eggs decreased rapidly, but the elimination time of the residue was long, and a low amount of residue was maintained for a long time, not being easy to clear. The residue of tylvalosin in eggs decreased rapidly; the residue had dropped to 2.5 μg/kg on the tenth day after drug withdrawal, which was lower than the detection limit of the national standard method GB31613.2-2021 [21]. Tylvalosin mainly remained in the yolk, and the residue in the albumen was very low, with the highest residue being only 6 μg/kg. The contents of fat-soluble drugs in yolk are much higher than those in albumen, while the contents of water-soluble drugs in albumen are higher than those in egg yolk [17,22]. The distributions of drugs in white and yolk also depend on the process of egg formation [23].

The maximum residue limit for erythromycin A in eggs according to GB 31650-2019 is 50 μg/kg. The erythromycin A residue in whole eggs must not fall below 50 µg/kg until the fourth day after withdrawal. The erythromycin A residue in the albumen peaked faster than that in the yolk, while the residue in the yolk persisted longer than that in the albumen. Throughout the test period, the residues of tylosin A and tyvalosin in eggs were below the MSLs of 300 and 200 µg/kg allowed for by GB 31650-2019, respectively, and the residues of erythromycin A, tylosin A and tylvalosin in all the eggs in the control group were below the corresponding detection limits of the method.

#### 3.2.3. Elimination of Lincomycin and Its Main Metabolites

As shown in Figure S4, lincomycin (0.5 μg/kg) was detected in eggs on the first day of lincomycin administration. From second to tenth day, the lincomycin residue in eggs increased rapidly, peaking on the eleventh day (225 μg/kg). After drug withdrawal, the lincomycin residue in eggs decreased rapidly. On the fifth day, the residue in whole eggs had fallen below 50 μg/kg (47 μg/kg), and on the eighth day, the residue in egg yolk had fallen below 50 μg/kg (33 μg/kg).

#### 3.2.4. Elimination of Tiamulin and Its Main Metabolites

As shown in Figure S4, tiamulin residues were detected on the second day in the tiamulin administration group. The tiamulin residues in eggs increased rapidly during the second and third days and peaked on the fourth. After drug withdrawal, the tiamulin residues in eggs decreased rapidly.

### 3.3. WDTs and MRLs

The WDT refers to the interval between the cessation of drug administration to animals and the approval for their slaughter or the marketing of their products [24]. The maximum residue limits (MRLs) of the seven antibiotics in eggs are stipulated in GB 31650-2019, as listed in Table 5. If the residues of these drugs in eggs are higher than their MRLs, it may lead to toxicity, allergic reactions, and the emergence of bacterial strains resistant to antibiotics [25].

**Figure 2:**
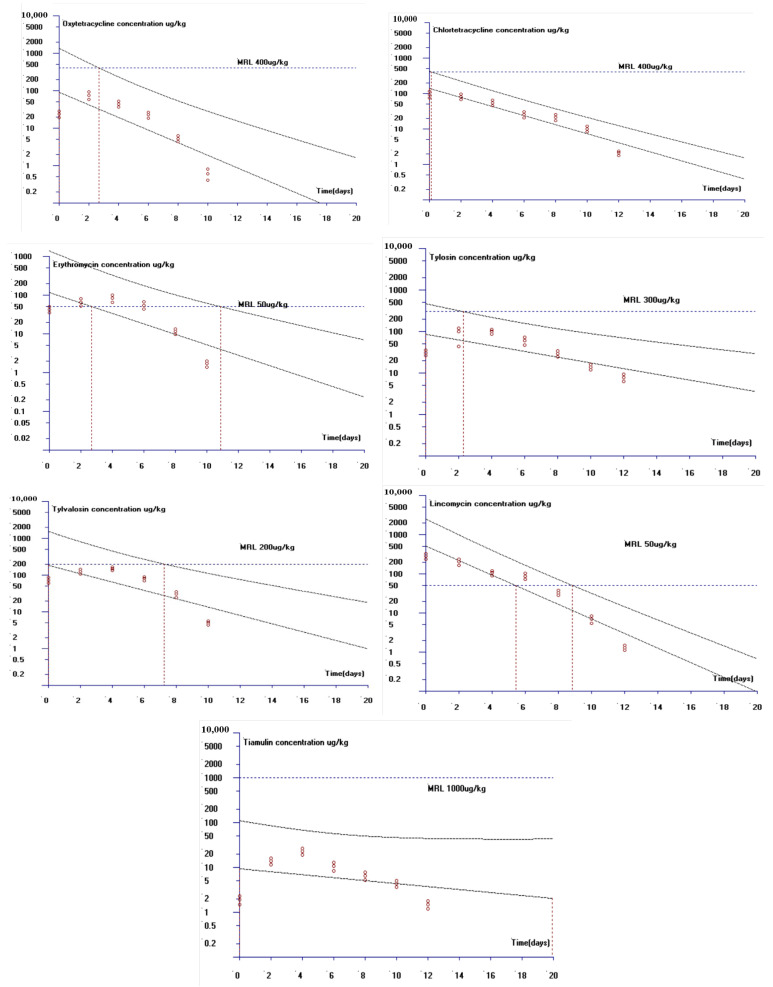
Calculation of the withdrawal times for oxytetracycline, chlortetracycline, erythromycin, tylosin, tylvalosin, lincomycin, and tiamulin to keep them below the MRLs (with 95% tolerance limits and 95% confidence intervals). Each circle represents an individual concentration of drug measured per day. The black lines are the linear regression and 95% tolerance limit with 95% confidence. The blue line represents the maximum residue limit in GB 31650-2019.

Referring to the MRLs of the antibiotics in eggs formulated in GB 31650-2019, the theoretically recommended WDTs of oxytetracycline, chlortetracycline, erythromycin, tylosin, tylvalosin, lincomycin, and tiamulin in Taihang chicken eggs are 2.8, 0.3, 11, 2.4, 7.4, 8.9, and 0 days, respectively, as calculated using the withdrawal time calculation software WT1.4, as shown in Figure 2. Therefore, it is recommended that the WDTs of oxytetracycline, chlortetracycline, erythromycin, tylosin, tylvalosin, lincomycin, and tiamulin in Taihang chicken eggs be 3, 1, 11, 3, 8, 9, and 0 days, respectively, in practice.

## 4. Discussion

This study aims to evaluate the WDI of seven antibiotics in eggs to protect the safety of food eggs. A revision of the WDT based on the elimination of antibiotic residues in specific animal species should be considered [26]. In different antibiotic administration groups, higher concentrations were observed in the egg yolk, which was consistent with the results of Hy-line hens [27]. The egg yolk was identified as the target tissue for estimating the withdrawal time periods (WDTs) of these seven drugs. The reason is that the residual concentrations of these seven drugs in the egg yolk, as an egg component, are relatively higher and the depletion speed is relatively slower. These differences are caused by multiple factors, such as the physicochemical properties of drugs, the physiological characteristics of hens, and the egg formation process [18]. When oxytetracycline and tylosin were continuously fed to Leghon hens [23], respectively, it was found that with the extension of the medication time, the contents of the two antibiotics in both the egg white and the egg yolk increased, and the concentration in the egg yolk was the highest. Based on the measured data, the egg yolk was determined as the target tissue. Considering the maximum residue limits (MRL), the withdrawal periods for oxytetracycline and tylosin were set at 9 days and 3 days, respectively. This is consistent with the experimental results of the oxytetracycline and tylosin groups in this study. Due to the differences in experimental animals, the withdrawal periods for oxytetracycline and tylosin formulated in this study are both 3 days. The passing rate of Silky fowls [26] in the national agricultural product quality and safety monitoring conducted by the Ministry of Agriculture and Rural Affairs has consistently remained relatively low among livestock and poultry products.

Sara Bogialli [28] administered 1.5 g/L of 20% erythromycin to hens for 5 consecutive days and collected eggs at the end of the administration period. The concentration peaked on the first day after the end of administration (day 6, 135 ng/g) and then decreased rapidly to 20 ng/g by day 8, which is well below the EU MRL (150 ng/g). These results are consistent with the trends in this study. However, in this study, the egg yolk was taken as the basis for estimating the withdrawal period, and the withdrawal period for erythromycin was set 11 days. Gbylik-Sikorska [29] fed lincomycin to laying hens at 20 mg/kg bw for 5 consecutive days, a dose lower than the MRL for eggs (50 mg/kg). The maximum concentration of lincomycin in albumen reached 47.2 mg/kg on the second day of administration, while the maximum concentration of lincomycin in whole eggs reached 43.2 mg/kg on the fourth day of administration. The results were also consistent with the trends in this study. However, the withdrawal period for lincomycin set in this study was 9 days. The elimination pattern of antibiotics in different tissues is also an important factor influencing the establishment of the withdrawal period.

Different feeding regimes, different animal breeds, and other factors may alter the metabolic elimination cycles of drugs. Due to different dosages and intervals of oral drugs, different researchers have reported different drug residue concentrations [30]. In addition, the intestine of Taihang chickens is 0.2–0.3 cm longer than that of ordinary laying hens, and the digestive power is stronger [31]. The structure and adaptability of the intestine may affect the absorption and retention times of drugs, and there are differences in drug metabolism. At present, the withdrawal period for drugs prescribed by the government is within one week. The metabolism of these drugs in Taihang is faster and the times are significantly shorter than those in other laying hens, which undoubtedly causes egg wastage. Therefore, it is necessary to establish withdrawal periods for specific breeds of hens [26,27]. In addition, testing only for residues in the whole egg may mislead the assessor’s judgement of the residue due to a dilution effect of one matrix on another matrix. Because egg yolk and albumen are separated in highly processed foods, it is necessary to analyse the residual concentrations in albumen and egg yolk and the associated toxic potential [20].

In this study, Taihang chickens were medicated through oral feeding, and the levels of residue markers were detected using UPLC-MS/MS. Throughout the study period, the residues of oxytetracycline, chlortetracycline, tylosin, tylvalosin, and tiamulin in the eggs were all below the MRLs allowed for in GB 31650-2019. The European Medicines Evaluation Agency’s Committee for Veterinary Medicinal Products recommends that a drug’s WDT be set as the point at which the drug’s residue falls below the specified MRL [32,33].

Throughout the experimental period, the residual amounts of oxytetracycline, chlortetracycline, tylosin, tylvalosin, and tiamulin in eggs were all lower than the maximum residue limits allowed for in GB 31650-2019. It is recommended that the withdrawal period for tiamulin be set at 0 days. However, based on where the upper limits of the 95% confidence intervals for oxytetracycline, chlortetracycline, tylosin, and tylvalosin fall below the MRLs, the theoretical recommended WDTs would be 2.8, 0.3, 2.4, and 7.4, respectively. It is recommended that the WDTs for oxytetracycline, chlortetracycline, tylosin, and tylvalosin in Taihang chicken eggs be set at 3 days, 1 day, 3 days, and 8 days, respectively. On the seventh day after withdrawal, the residue of erythromycin in the egg yolk fell below the MRL of 50 μg/kg allowed for in GB 31650-2019, but according to where the upper limit of the 95% confidence interval of erythromycin fell below the MRL, the theoretical recommended WDT would be 11. It is recommended that the WDT for erythromycin in Taihang chicken eggs be 11 days. On the eighth day after drug withdrawal, the residue of lincomycin in whole eggs fell below the MRL of 50 μg/kg allowed for in GB 31650-2019, but according to where the upper limit of the 95% confidence interval of lincomycin fell below the MRL, the theoretical recommended WDT would be 8.9. It is recommended that the WDT for lincomycin in Taihang chicken eggs be 9 days. This will not only avoid the wastage of eggs, but also ensure effective drug use and food safety. It is hoped that the results of this study will provide data support for the formulation of the WDTs of antimicrobial drugs for Taihang chickens; at the same time, it will provide technical references for responding to the comprehensive management activities for veterinary antimicrobial drugs carried out by the Ministry of Agriculture and Rural Affairs of China, standardising the use of drugs and ensuring the development of the Taihang chicken industry.

## 5. Conclusions

This study has confirmed the residue elimination status of seven antibiotics in eggs after oral administration to laying hens and established the withdrawal time. The LC-MS/MS method verified in this study was adopted to evaluate the drug residue levels in albumen, yolk, and whole eggs. The egg yolk was identified as the target tissue for estimating the withdrawal time periods (WDTs) of these seven drugs. The recommended withdrawal time of tiamulin in the eggs of Taihang chickens was 0 days. The recommended withdrawal times of oxytetracycline, chlortetracycline, erythromycin, tylosin, tylvalosin, and lincomycin were 3, 1, 11, 3, 8, and 9 days, respectively.

## Figures and Tables

**Table 1:** Dosage and method for each experimental group.

Group	Formulation	Administration
Oxytetracycline	50% oxytetracycline hydrochloride soluble powder	Mixed feeding: 1.0 g of 50% oxytetracycline hydrochloride soluble powder was added to each kilogramme of basal ration for 5 days.
Chlortetracycline	20% chlortetracycline hydrochloride soluble powder	Mixed feeding: 4.0 g of 20% chlortetracycline hydrochloride soluble powder was added to each kilogramme of basal ration for 5 days.
Erythromycin	5% erythromycin thiocyanate soluble powder	Mixed feeding: 5.0 g of 5% erythromycin thiocyanate soluble powder was added to each kilogramme of basal ration for 5 days.
Tylosin	50% tylosin tartrate soluble powder	Mixed feeding: 2.0 g of 50% tylosin tartrate soluble powder was added to each kilogramme of basal ration for 5 days.
Tylvalosin	20% tylvalosin tartrate soluble powder	Mixed feeding: 3.0 g of 20% tylvalosin tartrate soluble powder was added to each kilogramme of basal ration for 5 days.
Lincomycin	5% lincomycin hydrochloride soluble powder	Mixed feeding: 6.0 g of 5% lincomycin hydrochloride soluble powder was added to each kilogramme of basal ration for 10 days.
Tiamulin	45% tiamulin fumarate soluble powder	Mixed feeding: 1.11 g of 45% tiamulin fumarate soluble powder was added to each kilogramme of basal ration for 3 days.

**Table 2:** Detection conditions for seven kinds of antibiotics.

Analyte	Retention Time/min	Precursor Ion (m/z)	Daughter Ion (m/z)	Collision Voltage/V	Cone Voltage/V
Oxytetracycline	2.59	460.8	200.7 425.8	44 20	2
Chlortetracycline	3.30	478.8	153.7 443.8	26 20	34
Erythromycin A	3.48	734.2	158.1 576.2	30 18	30
Tylosin A	3.52	916.2	145.1 174.1	35 35	20
Tylvalosin	3.79	1042.7	109.1 174.1	45 45	30
Lincomycin	2.13	407.0	126.2 359.2	22 16	30
Tiamulin	3.61	494.4	119.0 192.1	25 19	10

**Table 3:** Methodological parameters.

Residual Marker	Regression Equation	R^2^ Correlation Coefficient	LOD (µg/kg)	LOQ (µg/kg)
Oxytetracycline	y = 3517.59x − 2130.43	0.9958	0.5	2.0
Chlortetracycline	y = 3242.91x − 1900.16	0.9969	0.5	2.0
Erythromycin A	y = 3357.56x − 2557.44	0.9951	0.5	2.0
Tylosin A	y = 1040.77x − 327.193	0.9977	1	5.0
Tylvalosin	y = 11,357.9x − 2472.89	0.9993	0.2	1.0
Lincomycin	y = 28,955.9x + 3765.47	0.9993	0.2	1.0
Tiamulin	y = 67,247.6x + 11453	0.9978	0.2	1.0

**Table 4:** The accuracy, precision, CCα, and CCβ of the method for the determination of seven kinds of antibiotics in whole egg, albumen, and yolk.

Analyte	Matrix	Spiked Concentration (μg/kg)	Recovery (%) (*n* = 6)	Intra-day RSD (%) (*n* = 6)	Inter-day RSD (%) (*n* = 6)	CCα (μg/kg)	CCβ (μg/kg)
Oxytetracycline	Whole egg	10	77	3.2	4.3	0.305	0.511
	Yolk	10	75	3.5	3.7	0.288	0.488
	Albumen	10	75	3.6	2.7	0.279	0.457
Chlortetracycline	Whole egg	10	71	2.5	2.0	0.304	0.493
	Yolk	10	74	4.0	3.3	0.253	0.486
	Albumen	10	73	3.8	2.9	0.266	0.442
Erythromycin A	Whole egg	10	78	2.6	3.5	0.331	0.476
	Yolk	10	81	1.8	3.8	0.345	0.439
	Albumen	10	80	2.9	3.1	0.324	0.469
Tylosin A	Whole egg	10	72	2.3	2.8	0.319	0.457
	Yolk	10	70	2.6	2.2	0.247	0.472
	Albumen	10	71	3.7	3.6	0.322	0.432
Tylvalosin	Whole egg	10	82	3.5	4.5	0.318	0.421
	Yolk	10	81	3.1	4.1	0.316	0.414
	Albumen	10	85	1.9	2.6	0.308	0.407
Lincomycin	Whole egg	10	90	2.2	2.8	0.302	0.422
	Yolk	10	88	2.7	3.2	0.254	0.418
	Albumen	10	92	2.6	2.4	0.337	0.434
Tiamulin	Whole egg	10	98	2.2	1.8	0.266	0.491
	Yolk	10	97	3.3	1.9	0.238	0.503
	Albumen	10	99	3.8	2.3	0.223	0.401

**Table 5:** GB31650-2019 MRLs and withdrawal times.

Veterinary Drug	31650 MRLs	CAC MRL	EU MRL	Chinese Veterinary Pharmacopoeia Withdrawal Time	Recommended WDT in Taihang Chicken Eggs
Oxytetracycline	400 μg/kg	400 μg/kg	200 μg/kg	5 days	3
Chlortetracycline	400 μg/kg	400 μg/kg	200 μg/kg	7 days	1
Erythromycin	50 μg/kg	50 μg/kg	150 μg/kg	3 days	11
Tylosin	300 μg/kg	300 μg/kg	200 μg/kg	1 days	3
Tylvalosin	200 μg/kg		200 μg/kg	5 days	8
Lincomycin	50 μg/kg		50 μg/kg	5 days	9
Tiamulin	1000 μg/kg		1000 μg/kg	5 days	0

## Data Availability

All the datasets collected and analysed during the current study are available from the corresponding author upon request; the availability of the data are restricted to investigators based at academic institutions.

## Supplementary Materials

The following supporting information can be downloaded at: https://www.mdpi.com/article/10.3390/ani14243701/s1, Figure S1: Quantitative ionogram of egg matrix spiked with seven antibiotic standards at 50 μg/kg; Figure S2: Residue depletion curves of Oxytetracycline (A) and Chlortetracycline (B) in whole eggs, albumen and yolks; Figure S3: Residue depletion curves of Erythromycin A (C), Tylosin A (D) and Tylvalosin (E) in whole eggs, albumen and yolks; Figure S4: Residue depletion curves of Lincomycin (F) and Tiamulin (G) in whole eggs, albumen and yolks.
