# Supplementary material for: Residue Elimination Patterns and Determination of the Withdrawal Times of Seven Antibiotics in Eggs of Taihang Chickens
Source: Animals (Basel). 2024 Dec 22;14(24):3701. doi: 10.3390/ani14243701 (PMC11672755; doi:10.3390/ani14243701)
Supplement: Supplementary file 1 [file animals-14-03701-s001.zip › animals-3296137-supplementary.pdf]

supplementary material

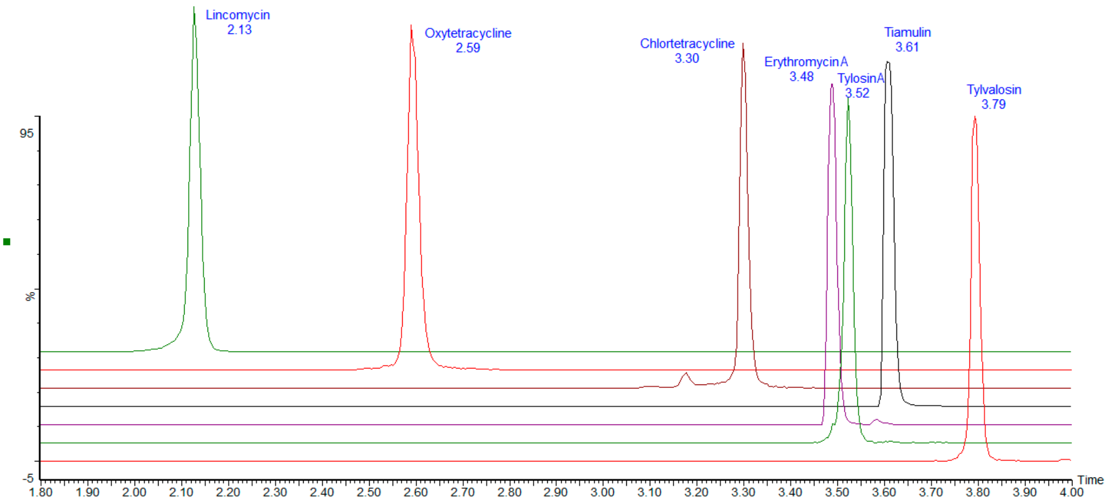

Figure S1: Quantitative ionogram of egg matrix spiked with seven antibiotic standards at 50 µg/kg.

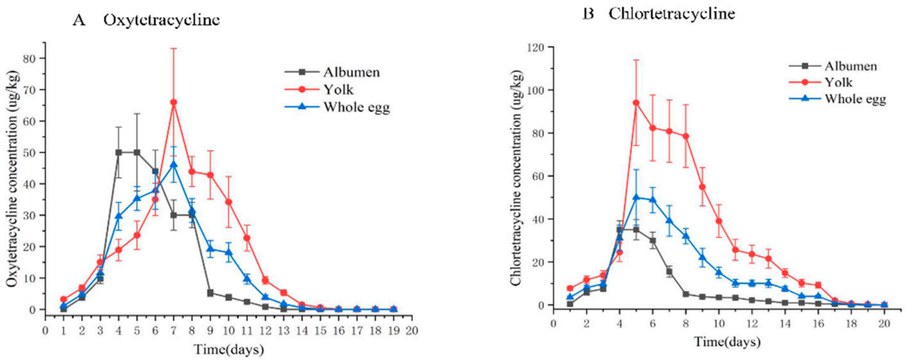

Figure S2: Residue depletion curves of Oxytetracycline (A) and Chlortetracycline (B) in whole eggs, albumen and yolks.

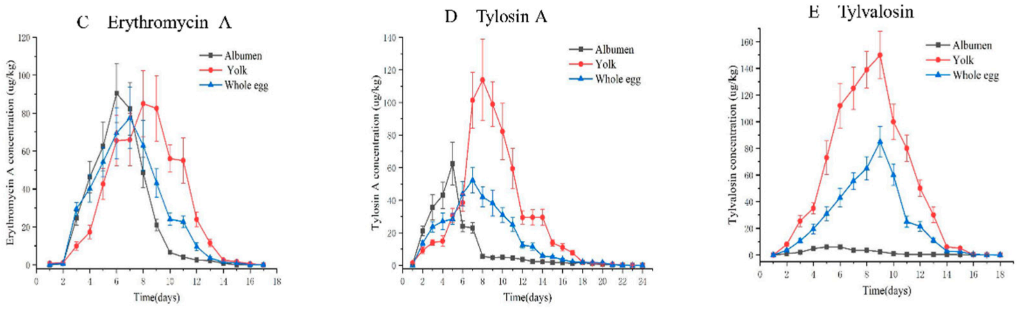

Figure S3: Residue depletion curves of Erythromycin A(C) , Tylosin A (D) and Tylvalosin (E)in whole eggs, albumen and yolks.

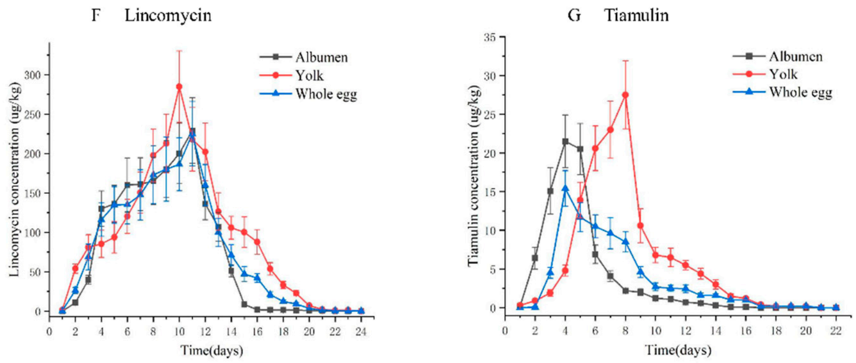

Figure S4: Residue depletion curves of Lincomycin (F) and Tiamulin (G) in whole eggs, albumen and yolks.
